# The Arterial Switch in the Modern Era: So Far, So Good∗

**DOI:** 10.1016/j.jacadv.2023.100429

**Published:** 2023-07-28

**Authors:** David J. Barron

**Affiliations:** Temerty Faculty of Medicine, Division of Cardiovascular Surgery, The Hospital for Sick Children, Toronto, Ontario, Canada

**Keywords:** arterial switch, outcomes, transposition

**Figure:**
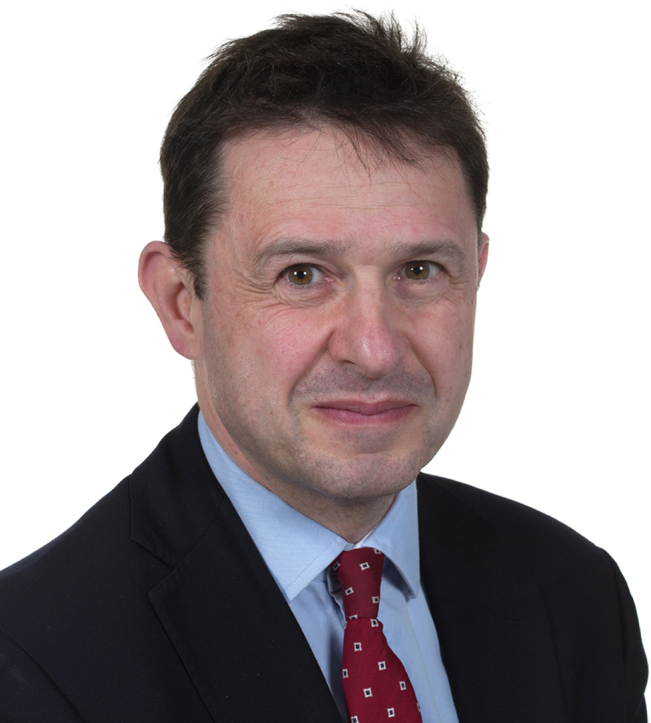

The arterial switch procedure is one of the triumphs of neonatal cardiac surgery, and this extensive report from the United Kingdom registry provides further evidence of its success and reproducibility at a national level in the modern era.

The paper from Dorobantu et al^1^ in this issue of *JACC: Advances* highlights the value of national registry data, especially in view of the unique strengths of the National Congenital Heart Disease Audit in the United Kingdom—which is mandatory data submission that captures every single cardiac surgical case in the country. Many of the multicenter registries and administrative databases that we look to as benchmarks for outcomes are voluntary submissions from selected institutions and may not always truly reflect the ‘real world.’ The data are further strengthened by the fact that every patient in the United Kingdom is traceable through every encounter with the health service, which has produced a detailed analysis of the ongoing health care needs of the entire population. The authors should be congratulated for such a detailed analysis in linking several national quality databases to track close to 1,800 patients over 17 years.

So, what does it tell us? There is no doubt that the outcomes on a national level are extremely good, consistent, and comparable with the best in the world. A hospital survival of 97.6% underlines the success of the procedure for a condition that was all but lethal in our grandparents’ generation. The success of the arterial switch is also emphasized by the low burden of care for the vast majority of cases after the first year of life. However, beyond these (valuable) findings, many of the questions around timing and risk factors are not really answered. It is frustrating that such a detailed database did not record coronary patterns, which have been repeatedly shown to be the greatest risk factor both for in-hospital mortality and early surgical reinterventions (almost all related to intramural coronaries or single coronary origin). Equally, great vessel relationship was not recorded and may have an important influence on late right ventricular outflow tract reinterventions. The preoperative ‘cardiac risk factors’ used by the authors were impaired ventricular function or pulmonary hypertension, which do not really address the key morphological issues of coronary patterns, commissural malalignment, and side-by-side great vessels that would most influence surgical technique. Although late deaths are very rare, the study does not identify the causes of death in these patients and what their anatomic risk factors might have been.

Timing of the switch has long been debated and it is important to note that late presentation (>3 weeks) is virtually unknown in this study and suggests that it has almost vanished from modern practice in developed countries. The median age at surgery of 9.5 days is slightly older than some recent studies (6.0 days in the Society for Thoracic Surgery database^2^), but there appeared to be no difference in outcome related to exact age at surgery—probably reflecting that with essentially no late presentations, outcomes are extremely good if surgery is performed before 3 weeks of age. There is no doubt that balloon atrial septostomy (BAS) can be life-saving, but the study is unable to untangle the overall role of BAS in management, probably because of the variable degree of naturally existing atrial communication in these patients. The fact that the use of BAS varies by 2.5-fold among centers suggests there is clearly varying institutional approach, probably with some favoring more routine BAS regardless of adequate mixing. Their matched group analysis supports their conclusion that selective use of BAS is preferable, and that primary switch when clinically feasible may minimize risk. Other findings are less easy to explain, and it is unclear why the need for renal replacement therapy was so high (14%) in the context of such good overall outcomes, nor why antenatal diagnosis was associated with worse outcomes, where it has previously been shown to reduce risk by allowing for planned delivery and pre-emptive management of hypoxia.

The study supports the growing evidence around reinterventions: firstly, that it is relatively uncommon and secondly, that it occurs in 2 distinct phases: 1) in the first year of life which includes most coronary reinterventions; and 2) in the 2 to 5 year age group which is most commonly branch pulmonary artery and RVOT interventions, more of half of which can be achieved in the cath lab. The unanswered question is the expected third phase of reintervention, which we are beginning to see in the older patients,^3^^,^^4^ and that is neo-aortic root dilatation and aortic regurgitation. Only by following these cohorts of patients for longer will we know the full story of reinterventions related to the switch.

As the 21st century progresses, we are increasingly dealing with the sequelae of the successes from the past—and we do not yet know what the future will hold for our populations of post-arterial switch in their 50s, 60s, and 70s. Just as we have learnt in adult repaired tetralogy of Fallot, we have discovered that the surgical techniques and postoperative course of these patients in their infancy have profound implications on their late functional status and need for reinterventions. As we move away from mortality analysis, the focus is on optimizing cardiovascular function and minimizing lifelong intervention. Robust and all-inclusive databases such as these will be key to learning these lessons and it is essential that they are well-designed from the outset to include all the key variables and operative details. One of the many challenges is that it will require ever greater analysis and accuracy of data to gain ever smaller nuggets of new knowledge. All praise to the data analysts!

## Funding support and author disclosures

The author has reported that he has no relationships relevant to the contents of this paper to disclose.
